# Resistant Maltodextrin Alleviates Dextran Sulfate Sodium-Induced Intestinal Inflammatory Injury by Increasing Butyric Acid to Inhibit Proinflammatory Cytokine Levels

**DOI:** 10.1155/2020/7694734

**Published:** 2020-09-16

**Authors:** Shilan Wang, Shiyi Zhang, Shimeng Huang, Zhenhua Wu, Jiaman Pang, Yujun Wu, Junjun Wang, Dandan Han

**Affiliations:** State Key Laboratory of Animal Nutrition, College of Animal Science and Technology, China Agricultural University, Beijing 100193, China

## Abstract

Inflammatory bowel disease (IBD), one kind of intestinal chronic inflammatory disease, is characterized by colonic epithelial barrier injury, overproduction of proinflammatory cytokines, and fewer short-chain fatty acids (SCFAs). The present study is aimed at testing the hypothesis that resistant maltodextrin (RM), a soluble dietary fiber produced by starch debranching, alleviated dextran sulfate sodium- (DSS-) induced colitis in mice. Female C57BL/6 mice with or without oral administration of 50 mg/kg RM for 19 days were challenged with 3% DSS in drinking water to induce colitis (from day 14 to day 19). Although RM could not reverse DSS-induced weight loss or colon shortening, it reduced inflammatory cell infiltration and epithelial damage in colon tissue, as well as the transfer of intestinal permeability indicators including serum diamine oxidase (DAO) and D-lactic acid (D-LA). ELISA analysis indicated that RM significantly suppressed the increase of Th1 cytokines induced by DSS in the colon such as tumor necrosis factor-*α* (TNF-*α*) and interferon-*γ* (IFN-*γ*). The levels of proinflammatory cytokines interleukin-1*β* (IL-1*β*), IL-17, and IL-8 in the DSS group were significantly higher than those in the control group and RM group, but no significant difference was observed in the RM-DSS group compared with the RM group. Interestingly, IL-10 levels of the DSS group were significantly higher than those of the other groups. With respect to SCFAs, DSS administration significantly decreased the concentration of faecal butyric acid while the RM-DSS group showed a tendency to increase (*P* = 0.08). In general, RM alleviated dextran sulfate sodium-induced intestinal inflammation through increasing the level of butyric acid and subsequently inhibiting the expression of proinflammatory cytokines.

## 1. Introduction

Inflammatory bowel disease (IBD), including ulcerative colitis (UC) and Crohn disease (CD), is one of the intestinal chronic inflammatory diseases [1]. The incidence and prevalence of IBD are on the rise worldwide [1]. Epithelial invasion of intestinal flora along with incomplete intestinal epithelial barriers is increasingly considered to have a causal relationship with IBD [2]. The gut inflammation occurring in patients with IBD is associated with excessive responses of Th1 or Th2 cells [3] and cytokines produced by Th17 cells [4]. It is also closely related to increased proinflammatory cytokines, e.g., tumor necrosis factor-*α* (TNF-*α*), interleukin-6 (IL-6), and interleukin-1*β* (IL-1*β*) [5]. In the mucosa and faeces of IBD patients, there were significantly fewer bacteria that can ferment fiber and produce short-chain fatty acids (SCFAs) than in healthy people [2].

As a global disease, the research to prevent of IBD is urgent. Antibiotics, prebiotics, live biotherapy, and faecal microbiota transplantation are considered to support the therapies for IBD [6]. Prebiotic is a one of major energy sources for the gut microbiome and thus can potentially change its composition in a beneficial way [7]. Additionally, previous research showed that gastrointestinal peristaltic disorders were associated with IBD [8]. Some dietary fibers are conducive to bowel movements [9] and positively affect IBD. The physical and chemical properties of fiber include fermentability, solubility, and viscosity, which will not only affect fermentation but also influence the therapeutic effects. According to the solubility, dietary fibers can be divided into insoluble fibers (cellulose, wheat bran, etc.) and soluble fibers (inulin, fructooligosaccharide, wheat dextrin, resistant maltodextrins, resistant starch, polydextrose, soluble corn fiber, etc.) [10, 11]. Intake of insoluble dietary fibers has the potential to decrease constipation, and soluble dietary fibers can reduce diarrhea to benefit IBD [10]. Besides, some soluble dietary fibers can be fermented by intestinal flora to produce short-chain fatty acids (SCFAs), especially butyric acid, which has anti-inflammatory properties and immunomodulatory functions [2, 9]. It has been previously observed that resistant dextrin, fructan-containing fiber, chitosan oligosaccharide, and inulin have beneficial effects on immune-mediated inflammation and can improve disease activity [7, 12–15].

Resistant maltodextrin (RM), a soluble dietary fiber [16] classified as resistant starch type V, is produced by debranching of the starch structure [17]. Fibersol-2 (a type of digest-resistant maltodextrin) showed anticancer activity *in vitro* [18] and is fermentable in the colon by colonic bacteria and produces short-chain fatty acid [17]. Fibersol-2 can also increase faecal *Bifidobacterium* populations and butyrate proportion [19]. Data from several studies suggest that isomaltodextrin (a highly branched alpha-glucan and a type of resistant starch) helps for anti-inflammation and benefits in not only preventing low-grade chronic systemic inflammation [20] but also alleviating intestinal inflammation [21]. Before dextran sulfate sodium (DSS) treatment, supplementing with resistant starch (RS) type 3 for 7 d can improve colonic lesions in rats, which is achieved through producing high levels of butyrate [22]. However, there has been no detailed investigation of the preventive effect of RM on the intestinal inflammation.

Moreover, a dextran sulfate sodium- (DSS-) induced colitis mouse model is widely used in pathophysiological studies, especially for the testing of drug and nutritional therapies of IBD [23]. Given the fact that food first enters the digestive system, it can be said that diet plays a part in the prevalence of IBD [24]. Dietary recommendations for IBD prevention and management are few and not based on evidence [25]. Therefore, the main purpose of this study was to assess the preventive effects of RM on IBD and explore the mechanisms.

## 2. Materials and Methods

All experimental protocols were carried out with the approval of the China Agricultural University Animal Care and Use Committee (AW10099102-1, Beijing, China).

### 2.1. Resistant Maltodextrin

Fibersol-2 was purchased from Matsutani Chemical Industry Co., Ltd., which is a kind of resistant maltodextrin.

### 2.2. Animals and Treatment

Female C57BL/6J mice (7 weeks old) were purchased from SPF Biotechnology Co., Ltd. (Beijing, China) and went through a one-week adaptation period. Animals were housed at 22-25°C with a 12 h light/12 h dark cycle. Standard chow and water were provided ad libitum [23].

**Figure 1:**
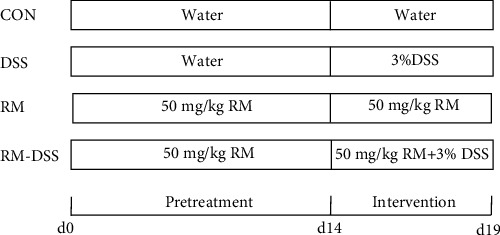
Animal model experimental procedure. RM was administered for the whole period. From day 14 to day 19, DSS (3% (*w*/*v*)) was added to drinking water to induce colitis. *n* = 10 mice/group.

All of the mice were randomly divided into four groups (*n* = 10/group): (i) control group (CON) was provided water; (ii) DSS group was provided 3% DSS (*w*/*v*) solution with distilled water; (iii) resistant maltodextrin group (RM) was provided RM (50 mg/kg body weight/day) dissolving in 100 *μ*L PBS by gavage; and (v) RM-DSS group was provided with 3% DSS (*w*/*v*) solution with distilled water and RM (50 mg/kg body weight/day) dissolving in 100 *μ*L PBS by gavage. Referring to some published articles [20, 21], the dose of resistant maltodextrin was chosen based on a pre-experiment, which included two doses (50 mg/kg body weight/day and 100 mg/kg body weight/day). RM was administered for 19 days. On the 14th day of the study, DSS (3% (*w*/*v*), molecular weight 36−50 kDa (MP Biomedical, Solon, OH, USA)) was added to drinking water to induce colitis and continued for the next 5 days [23]. The experimental procedure is shown in Figure 1.

### 2.3. Assessment of Colitis

During DSS treatment, the changes of the body weight, stool consistency, and faecal blood score of mice were recorded every day. The detail score standards are provided in Table 1. The disease activity index (DAI) score was defined as the sum of scoring from weight loss (%), stool consistency, and faecal blood content [26]. The length of the colon was recorded at the end of the study.

### 2.4. Biochemical Assays

All mice were sacrificed on the 19th day. The eyeballs were extracted under anesthesia, and blood was collected from the mice. Serum was separated and stored at -80°C for further experiments. Diamine oxidase (DAO) and D-lactate (D-LA) content was detected by ELISA kits (Nanjing Jiancheng Bioengineering Institute, Nanjing, China).

### 2.5. Histological Analysis

The proximal colon tissues were collected and stored in 4% paraformaldehyde solution, dehydrated, and paraffin-embedded. Sections (5 mm) were stained with haematoxylin and eosin and scored based on a previous study [5] (Table 2).

### 2.6. Cytokine Levels

On the 19th day, the proximal colon tissues were collected and frozen in liquid nitrogen for cytokine analysis. Frozen colon samples were stored at -80°C for subsequent experiments. The protein concentration was determined using the Pierce™ BCA Protein Assay Kit according to the instruction of the manufacturer. The concentrations of interleukin tumor necrosis factor-*α* (TNF-*α*), interferon-*γ* (IFN-*γ*), interleukin-1*β* (IL-1*β*), IL-10, IL-17, and IL-8 were detected according to the ELISA kit instruction designed by the Nanjing Jiancheng Bioengineering Institute (Nanjing, China).

### 2.7. Lactic Acid and Short-Chain Fatty Acid (SCFA) Concentrations

The stool samples of each mouse were collected in a sterile tube and stored at −80°C immediately. Ion chromatography was used to determine faecal lactic acid and SCFA concentrations according to a previous literature [27]. Briefly, approximately 30 mg faeces was weighed and diluted. The two-step dilution method was adopted, and the final solution equaled to 800-fold dilution. After ultrasonic wave and centrifugation, supernatants were filtered through a 0.22 *μ*m syringe membrane filter to remove the interference, and then, a 25 *μ*L sample was delivered to the system.

### 2.8. Statistics

The results were presented as the mean ± standard error of the mean (SEM). Data were analysed using one-way analysis of variance (ANOVA), followed by Tukey's multiple-comparison (GraphPad Prism version 8.0, San Diego, CA, USA). The statistic unit of each parameter was 10 samples per treatment. *P* < 0.05 was considered statistically significant, and 0.05 < *P* < 0.1 was considered a statistical trend.

## 3. Results

### 3.1. Effect of RM on the Development of DSS-Induced Colitis in Mice

**Figure 2:**
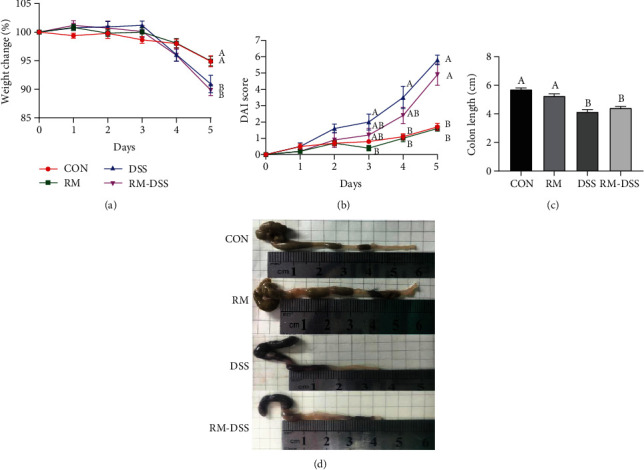
Symptoms of DSS-induced colitis: (a) body weight change, (b) disease activity index (DAI), (c) colon length, and (d) macroscopic pictures of colons. The data are mean ± SEM of *n* = 10 mice/group. Without the same letter indicated *P* < 0.05, which means there were significant differences between two groups. CON: group provided water; DSS: group provided 3% DSS (*w*/*v*) solution with distilled water; RM: group provided resistant maltodextrin (50 mg/kg body weight/day) dissolving in 100 *μ*L PBS by gavage; RM-DSS: group provided with 3% DSS (*w*/*v*) solution with distilled water and RM (50 mg/kg body weight/day) dissolving in 100 *μ*L PBS by gavage.

As shown in Figure 2, the induction of colitis with 3% (*w*/*v*) DSS for 5 days caused a significant body weight loss of mice in the DSS group and the RM-DSS group in the end compared with the CON and RM groups, respectively (*P* < 0.05) (Figure 2(a)). On the 3rd day after DSS treatment, DAI scores were higher in the DSS group (*P* < 0.05, DSS vs. RM) than in the RM-DSS group (*P* > 0.05, RM-DSS vs. RM). On the 4th day, DAI scores of the DSS group were significantly higher than those of the groups without DSS challenge (CON and RM groups) (*P* < 0.05), while there was no statistical difference between RM-DSS and groups without DSS challenge (CON and RM groups). On the 5th day, the DSS and RM-DSS groups showed higher DAI scores than the CON and RM groups (*P* < 0.05), respectively (Figure 2(b)). The colon length of the DSS and RM-DSS groups was significantly shorter than that of the CON and RM groups. There was no significant difference between the DSS and RM-DSS groups (Figure 2(c)). Although orally receiving resistant maltodextrin did not relieve the weight loss and colon shortening induced by DSS, it did delay the rise of DAI on the 3rd day and did not prevent the increased DAI score on the 4th and 5th days.

### 3.2. Effect of RM on Colon Histological and Morphological Damage in DSS-Treated Mice

**Figure 3:**
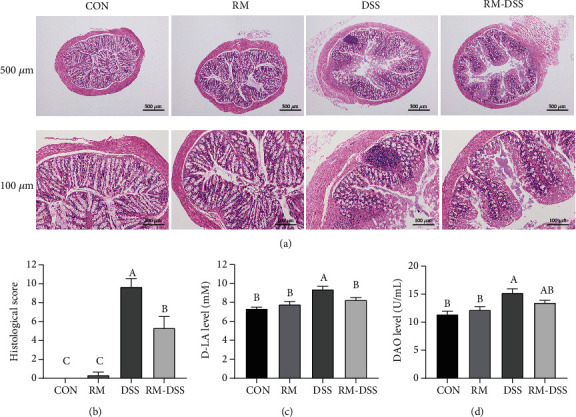
Effect of RM on histological and morphological damage in DSS-treated mice: (a) histological examination (the scale bars are 500 *μ*m and 100 *μ*m, individually), (b) colonic histological score, (c) D-LA, and (d) DAO. The data are shown by mean ± SEM of *n* = 10 mice/group. Different letters indicated *P* < 0.05, which means there were significant differences between the two groups. CON: group provided water; DSS: group provided 3% DSS (*w*/*v*) solution with distilled water; RM: group provided resistant maltodextrin (50 mg/kg body weight/day) dissolving in 100 *μ*L PBS by gavage; RM-DSS: group provided with 3% DSS (*w*/*v*) solution with distilled water and RM (50 mg/kg body weight/day) dissolving in 100 *μ*L PBS by gavage.

To further assess the symptoms of DSS-induced colitis, we evaluated the colon histological and morphological damage from three aspects, including loss of epithelial surface, destruction of the crypt, and infiltration of inflammatory cells. The haematoxylin-eosin staining showed that colon ulceration can be seen severely in the groups treated with DSS, and all of the crypts and epithelium surfaces were destructed. A large number of inflammatory cells were infiltrated in DSS group compared with CON group. The RM-DSS group displayed more intact intestinal epithelium and crypts with fewer neutrophil and monocyte infiltration compared with the DSS group (Figure 3(a)).

D-Lactate (D-LA) and diamine oxidase (DAO) can reflect the integrity and damage degree of the intestinal mechanical barrier. As shown in Figure 3, the serum D-LA level in the DSS group was significantly higher than the other three groups (*P* < 0.05). It means RM oral treatment inhibited significant increase of serum D-LA levels which is caused by DSS addition (Figure 3(c)). The levels of DAO in the DSS group were significantly higher than those in the control group (*P* < 0.05), while there was no significant difference between the CON group and the RM-DSS group (Figure 3(d)). It indicated RM alleviated intestinal barrier damage brought by DSS.

### 3.3. Effects of RM on Production of Inflammatory Cytokines in DSS-Treated Mice

**Figure 4:**
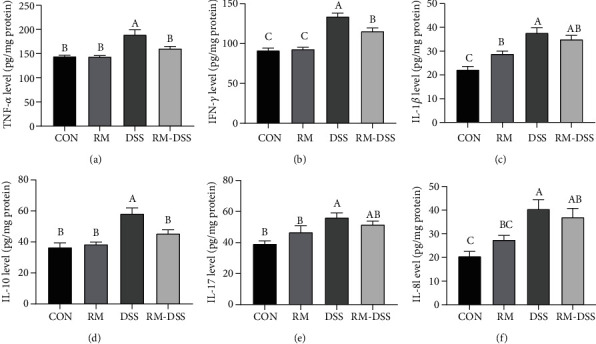
Effect of RM on the inflammatory cytokine production in the colons of mice with dextran sulfate sodium- (DSS-) induced chronic colitis. Protein was isolated from the colon tissues for ELISA analysis of TNF-*α* (a), IFN-*γ* (b), IL-1*β* (c), IL-10 (d), IL-17 (e), and IL-8 (f) levels. The data are mean ± SEM of *n* = 10 mice/group. Without the same letter indicated *P* < 0.05, and there were significant differences between the two groups. CON: group provided water; DSS: group provided 3% DSS (*w*/*v*) solution with distilled water; RM: group provided resistant maltodextrin (50 mg/kg body weight/day) dissolving in 100 *μ*L PBS by gavage; RM-DSS: group provided with 3% DSS (*w*/*v*) solution with distilled water and RM (50 mg/kg body weight/day) dissolving in 100 *μ*L PBS by gavage.

In order to investigate the intestinal inflammatory responses of DSS-treated mice with or without RM, we measured the cytokine levels, which are shown in Figure 4. The levels of the colon Th1 cytokine production (IFN-*γ* and TNF-*α*) in the DSS group were significantly higher than those in the control group, while for the RM-DSS group, RM supplement (50 mg/kg body weight/day) significantly inhibited IFN-*γ* and TNF-*α* production induced by DSS. The levels of IL-1*β*, IL-17, and IL-8 in the DSS group were significantly higher than those in both the control group and the RM group. However, these three cytokines did not show significant differences between the RM-DSS and RM groups (Figure 4). Interestingly, the IL-10 level of the DSS group was significantly higher than that of the other groups (Figure 4(d)).

### 3.4. Effect of RM on Lactic Acid and SCFAs in Mouse Faeces in DSS-Treated Mice

**Figure 5:**
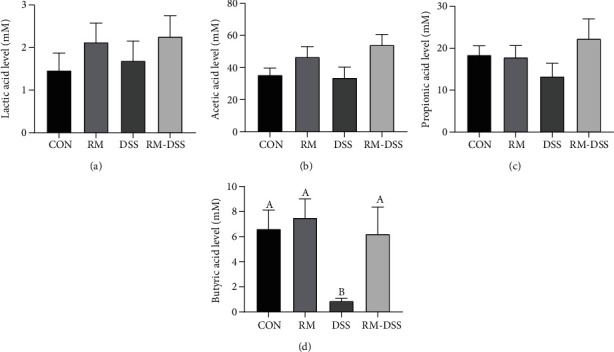
Effect of RM on faecal lactic acid and SCFAs of mice with dextran sulfate sodium- (DSS-) induced chronic colitis. Lactic acid (a), acetic acid (b), propionic acid (c), and butyric acid (d) levels. The data are mean ± SEM of *n* = 10 mice/group. Without the same letter indicated *P* < 0.05, and there were significant differences between the two groups. The *P* value between the DSS and RM-DSS groups was 0.08. CON: group provided water; DSS: group provided 3% DSS (*w*/*v*) solution with distilled water; RM: group provided resistant maltodextrin (50 mg/kg body weight/day) dissolving in 100 *μ*L PBS by gavage; RM-DSS: group provided with 3% DSS (*w*/*v*) solution with distilled water and RM (50 mg/kg body weight/day) dissolving in 100 *μ*L PBS by gavage.

Previous studies have shown that IBD patients display deficiency of short-chain fatty acids [2]. Besides, SCFAs can protect epithelial cells through inhibiting the production of proinflammatory cytokines *in vitro* [28]. In order to investigate whether RM reduced intestinal inflammation by increasing the concentrations of SCFAs, we measured lactic acid and SCFAs content in faeces, which is shown in Figure 5. Without considering the treatment group, the most abundant SCFA in the faeces was acetic acid, followed by propionic acid and butyric acid. The amount of butyric acid was significantly lower in the DSS group than in both the CON and RM groups (Figure 5(d)), while the RM-DSS group tended to increase it compared with the DSS group (*P* = 0.08). There were no significant differences of faecal lactate, acetate, and propionate levels among the four groups (Figure 5).

## 4. Discussion

In this study, we investigated the protective effects and underlying mechanism of RM on colitis in a DSS-challenged mouse model. Resistant starch and isomaltodextrin have shown the potential to reduce inflammation in the colon [29]. Resistant maltodextrin (RM), a soluble dietary fiber classified as resistant starch type V, will not be absorbed in the small intestine because of its debranching structure [30]. A previous study has even indicated anticancer activity of resistant maltodextrin *in vitro* [18]. In the present study, we found that RM oral administration could alleviate the DAI score increase induced by DSS, improve the intestinal histology damage, and significantly suppress the proinflammatory cytokines through increasing faecal butyric acid. These results illustrated that RM may possess an anti-inflammatory effect.

Intestinal epithelial barriers of IBD patients are incomplete [2], which also happens in the DSS-induced animal colitis model [23]. Biomarkers used to evaluate the intestinal injury are diamine oxidase (DAO) and D-lactic acid (D-LA) [30]. In order to clarify the severity of DSS-induced intestinal injury, we performed intestinal morphological analysis and histological scores and serum concentrations of DAO and D-LA measurement. Although adding RM did not improve the weight loss and colon shortening induced by DSS, it alleviated DAI increase through reducing inflammatory cell infiltration, epithelial damage in colon tissue, and serum DAO and D-LA. Dietary fermentation rice bran reduced intestinal inflammation by increasing SCFAs and may regulate the integrity of the tight junction barrier and intestinal homeostasis [31]. Isomaltodextrin administration increased expression of intestinal mucin 2, mucin 4, and the tight junction protein claudin 4 in mice challenged with lipopolysaccharide [20].

A previous study has also shown that the 2-week pretreatment of isomaltodextrin could not prevent weight loss or colon shortening induced by 5% DSS but did have potential capacity of anti-inflammation [21]. Diet containing 10% resistant starch showed anti-inflammatory and anticancer properties, protecting against colitis-associated colorectal cancer using a rat model induced by azoxymethane and 2% DSS [29]. This is probably because two-week preadministration of RM (50 mg/kg) is not long enough to protect mice challenged by 3% DSS. Longer pre-treatment should be considered prior to DSS [32]. However, some studies showed that inulin can exacerbate the severity of acute colitis in both low- and high-fat diets [12] and 10% flaxseed diet (including fermentable fiber) exacerbated DSS-induced colonic injury and inflammation [33]. Therefore, dietary fiber intake of IBD patients should take comprehensive consideration according to their daily diet and environment [9, 12, 34].

The gut inflammation occurring in patients with IBD is mainly characterized by increased proinflammatory cytokines [5]. The RM-DSS group (orally receiving RM 50 mg/kg body weight/day) significantly inhibited IFN-*γ* and TNF-*α* production, which indicated RM suppressed the excessive response of Th1 cells induced by DSS [3]. This result is consistent with previous studies that isomaltodextrin and chitosan oligosaccharide can reduce DSS-induced colonic inflammatory cytokines [13, 20, 21]. Based on the result of a higher level of IL-1*β*, IL-17, and IL-8 for the DSS group than the control group and the RM group, it can be inferred that orally receiving RM has the potential to inhibit proinflammation cytokines IL-1*β* and IL-17 produced by Th17 cells in the colon [35, 36]. This outcome is contrary to that of Li et al. who found levels of TNF-*α*, IL-17, and IL-1*β* in colon tissues decreased, while mRNA levels of these cytokines were significantly elevated in the colons of DSS-treated colitis mice [37]. It is important to bear in mind the possible bias of cytokine levels and its mRNA expression in these responses. Moreover, the upregulated level of IL-10 was detected in IBD patients, which was reacting to the chronic inflammation of the gastrointestinal tract [38]. In our study, IL-10 of the DSS group was significantly higher than that of the other three groups, which may be aimed at overcoming DSS-induced inflammation. Contrary to expectations, this study did not find a significant elevation of anti-inflammation cytokine IL-10 in the RM-DSS group. A possible explanation for this might be that the mice of the RM-DSS group restored more quickly to normal intestinal immune balance, followed by a decline of IL-10 levels in contrast to the DSS group, which is in agreement with the statement of Lee et al. [39]. Admittedly, the findings of previous researches showed that both probiotic and synbiotic including whole plant sugar cane fiber supplement significantly increased the levels of IL-10 [40, 41].

For IBD patients, there are significantly fewer bacteria in mucosa and faeces that can ferment fiber and produce short-chain fatty acids (SCFAs) than healthy people [2]. Butyrate is one of the most important energy sources for the colonic cell and has been reported for its anti-inflammation property [22]. Based on the result that the RM-DSS group tended to increase the butyric acid concentration compared with the DSS group (*P* = 0.08), it gave us a hint that RM may benefit mice through promoting butyric acid production. However, unexpectedly, there are no statistically significant differences for the butyrate level between the DSS and RM-DSS groups due to huge SEM. Study has shown that acetate can stimulate the expansion of Treg in the colon and increase the expression of anti-inflammatory cytokine IL-10 [42]. Although there was also no statistically significant difference for the acetate level between the DSS and RM-DSS group (*P* = 0.1157), higher levels of acetate could also be responsible for the preventive therapy of RM to gut inflammation induced by DSS. A previous study showed that a mixture of SCFAs was protected from DSS-induced colorectal cancer by improving colon inflammation and DAI as well as suppressing the expression of proinflammatory cytokines [43], which partly explained the role of RM in inhibiting proinflammatory cytokines. Another *in vitro* study showed that SCFAs (acetate, propionate, and butyrate) alleviated TNF-*α*-induced endothelial activation by inhibiting the production of proinflammatory cytokines (IL-6 and IL-8) [28], which is consistent with our results. Moreover, short-chain fatty acids may activate gut epithelium and immune cells through cell surface G-protein-coupled receptors GPR41, GPR43, and GPR109A [2, 35], which has been verified in a study of resistant starch [29].

Furthermore, resistant maltodextrin can be fermented by colonic bacteria, and after that, short-chain fatty acids are produced [17]. It will increase faecal *Bifidobacterium* populations and butyrate proportion [19]. Based on the character of resistant maltodextrin, it alleviates intestinal histological damage induced by DSS in mice through regulating inflammatory cytokines and butyric acid, which may be relevant with significant positive change of gut microbiota [29]. However, DSS carries a high negative charge provided by sulfate groups, which is toxic to colonic epithelial cells and can directly damage the integrity of the intestinal barrier [23]. Thus, it is not suitable enough to study intestinal flora [21]. Therefore, we suggest bringing other models in further studies to check if RM exhibits an anti-inflammation role through microbiota-dependent or microbiota-independent mechanisms [34].

## 5. Conclusions

In conclusion, resistant maltodextrin could increase the level of short-chain fatty acids in the colon, and butyric acid played an anti-inflammatory role to inhibit proinflammatory cytokines (IFN-*γ* and TNF-*α*), which finally presented as the intestinal morphological repair of DSS-induced colitis.

## Figures and Tables

**Table 1:** Scoring standards for the disease activity index (DAI)^1,2^.

Score	Weight loss (%)	Stool consistency	Faecal blood content
0	None	Normal	Normal
1	0-5		
2	5-10	Loose stool	Occult blood
3	10-20		
4	>20	Diarrheal	Haemorrhage/gross bleeding

^1^The DAI score was defined as the sum of scoring from weight loss (%), stool consistency, and faecal blood content. ^2^Refer to Park et al. with modification [26].

**Table 2:** Parameters and criteria of histological damage evaluation^1,2^.

Parameters	Score	Histological features
	0	No change
(1) Loss of epithelial surface	1	Localized and mild
(2) Destruction of crypt	2	Localized and moderate
(3) Infiltration of inflammatory cells	3	Localized and severe
	4	Extensive and moderate
	5	Extensive and severe

^1^The histological score was the sum of scoring from parameters (1), (2), and (3). ^2^Adapted from Ji et al. [5].

## Data Availability

The data used to support the findings of this study are available from the corresponding author upon request.
